# 473. Diabetes Mellitus as a Risk Factor for Cryptococcosis — a Multicenter Research Network Study

**DOI:** 10.1093/ofid/ofac492.531

**Published:** 2022-12-15

**Authors:** Vanessa Kung, Andrés F Henao-Martínez, Alex Tagawa, Matthew Kennis, Carlos Franco-Paredes, Lilian Vargas Barahona, Leland Shapiro, Daniel B Chastain

**Affiliations:** University of Colorado, Denver, Colorado; University of Colorado Anschutz Medical Campus, Aurora, Colorado; Children's Hospital Colorado, Denver, Colorado; University of Colorado, Denver, Colorado; Hospital Infantil de México, Federico Gómez, Denver, Colorado; MD, Denver, Colorado; Rocky Mountain Regional Veterans Affairs Medical Center, Denver, Colorado; University of Georgia College of Pharmacy, Albany, Georgia

## Abstract

**Background:**

Diabetes mellitus type 2 (DM2) is a common medical condition that increases the risk of bacterial infections, and is often present in patients with cryptococcosis. The role of DM2 as an independent risk factor for cryptococcosis is debatable. We aim to better characterize the natural history of cryptococcosis in patients with DM2 as their only comorbidity.

**Figure:**
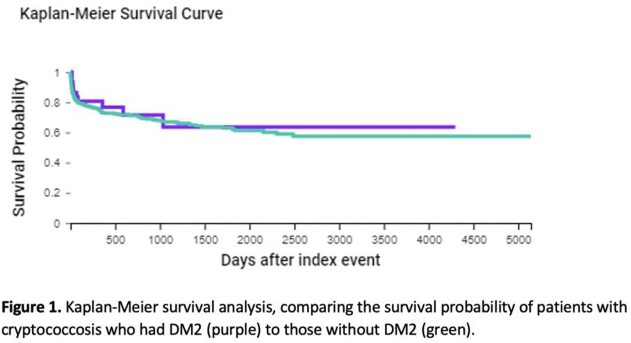

**Methods:**

We utilized TrinetX, a federal national network, to identify HIV-negative patients who had cryptococcosis without known risk factors. Demographic characteristics and outcomes were compared between patients with DM2 and those without DM2, who tested positive or had ICD based diagnoses of Cryptococcus infection within five years of diagnosis of DM2.

**Results:**

Sixty patients with DM2 (as the sole risk factor) and 707 patients without DM2 had cryptococcosis. Patients with DM2 and cryptococcosis were older (61 ± 13.6 years vs. 55.8 ± 16.2 years, p=0.0219), and more likely to be Hispanic or Latino (18% vs. 9%, p=0.023). They had higher rates of hypertension (77% vs 44%, p< 0.0001), cystic fibrosis (18% vs 1%, p< 0.0001), tuberculosis (18% vs 1%, p< 0.0001), and chronic kidney disease (33% vs 18%, p=0.0026). The mean HbA1c among patients with DM2 who developed cryptococcosis was 8.16 (SD 2.62). The most common sites of cryptococcus infection were pulmonary (56% vs 55%, p=0.8930) and cerebral (36% vs 40%, p=0.5589), in both groups. The two groups had similar mortality (20% vs 25.47%, p=0.3676) (Figure 1), and hospitalization rates (20% vs 31.3%, p=0.08). The overall annual cryptococcosis risk among HIV-negative patients with DM2 without any additional risk factors was 0.001%.

**Conclusion:**

Cryptococcosis occurs rarely in HIV-negative patients with DM2 and without additional risk factors. Hispanic or Latino ethnicity, uncontrolled hyperglycemia, and chronic kidney disease may increase the risk of cryptococcosis among patients with DM2. Cryptococcosis in patients whose only comorbidity is DM2 have as high of mortality as that seen with more established comorbitidies.

**Disclosures:**

**All Authors**: No reported disclosures.

